# Tissue Resident Foxp3^+^ Regulatory T Cells: Sentinels and Saboteurs in Health and Disease

**DOI:** 10.3389/fimmu.2022.865593

**Published:** 2022-03-11

**Authors:** Juyeun Lee, Dongkyun Kim, Booki Min

**Affiliations:** ^1^ Department of Cardiovascular and Metabolic Sciences, Lerner Research Institute, Cleveland Clinic, Cleveland, OH, United States; ^2^ Department of Microbiology and Immunology, Feinberg School of Medicine, Northwestern University, Chicago, IL, United States

**Keywords:** regulatory T cell, tissue residency, inflammation, homeostasis, Foxp3

## Abstract

Foxp3^+^ regulatory T (Treg) cells are a CD4 T cell subset with unique immune regulatory function that are indispensable in immunity and tolerance. Their indisputable importance has been investigated in numerous disease settings and experimental models. Despite the extensive efforts in determining the cellular and molecular mechanisms operating their functions, our understanding their biology especially *in vivo* remains limited. There is emerging evidence that Treg cells resident in the non-lymphoid tissues play a central role in regulating tissue homeostasis, inflammation, and repair. Furthermore, tissue-specific properties of those Treg cells that allow them to express tissue specific functions have been explored. In this review, we will discuss the potential mechanisms and key cellular/molecular factors responsible for the homeostasis and functions of tissue resident Treg cells under steady-state and inflammatory conditions.

## Introduction

The immune system is incessantly confronted by antigens derived not only from invading foreign pathogens but also from self. Highly efficacious processes are in place so that harmful pathogens are cleared by rapid yet indiscriminating innate immunity and by more sophisticated antigen-specific adaptive immunity. For those lymphocytes posing a risk of self-reactivity, they are effectively eliminated during the development within the primary lymphoid tissues. The elimination is incomplete though, thus some lymphocytes with potential self-reactivity still mature and seed the periphery. There comes a secondary screening procedure in the periphery to prevent unwanted activation of self-reactive lymphocytes, predominantly operated through regulatory T (Treg) cells (1, 2).

Treg cells are a subset of CD4 T lymphocytes that plays an indispensable role in maintaining immunity and tolerance. Treg cell development is regulated by the master transcription factor, Foxp3, which forms multiprotein complexes capable of activating or repressing gene transcription responsible for Treg cell differentiation and functions (3). Depending on the site of their generation, Treg cells are primarily divided into two distinct subsets, thymus-derived tTreg and peripherally induced pTreg cells. Foxp3 expression is acquired in developing T cells within the thymus, and the level of self-reactivity is thought to support the differentiation into tTreg lineage cells (4, 5). On the other hand, conventional CD4 T cells activated during immune responses may acquire Foxp3 expression under adequate conditions and become pTreg cells (inducible iTreg cells when generated *in vitro*) (5, 6). The chief importance of Treg cells in immunity and tolerance is well exemplified from the facts that Foxp3 deficiency or its functional mutation results in systemic lethal autoimmune inflammation (7, 8). Scurfy mice have a missense mutation in the Foxp3 gene due to 2-bp insertion, generating truncated Foxp3 protein and lacking Treg cells, and exhibit a fatal lymphoproliferative disease and premature death (9, 10). Likewise, deletion in Foxp3 gene causes scurfy-like phenotypes (11). Similarly, the immunodysregulation polyendocrinopathy enteropathy x-linked (IPEX) syndrome is a rare recessive disease with life-threatening multi-organ autoimmune inflammation, and mutations in the Foxp3 gene is responsible for the manifestation of the disease (12, 13). More than 70 mutations in the Foxp3 gene have been identified in IPEX syndrome, ranging from a single base substitution through deletion mutations (14, 15). Recapitulation of some of the IPEX mutation in mice results in the development of a fulminant autoimmune syndrome (16). Unlike the scurfy mutation or Foxp3 deletion in mice where Treg cell generation is severely impaired, some IPEX patients still display undisturbed Treg cell generation, suggesting functional defects of Treg cells (17). The contribution of tTreg and pTreg cells to the immunity and tolerance is generally overlapping, although there is evidence that pTreg cells may play an additional role in maintaining inflammation and tolerance especially at the mucosal tissues (18, 19). Indeed, deletion of the conserved noncoding sequence-1 (CNS-1) of the Foxp3 gene causes severe defects in TGFβ-dependent Foxp3 expression *in vitro* (i.e., iTreg cells) without affecting thymic generation of tTreg cells (20). These animals spontaneously develop Th2 type inflammation at mucosal sites (20, 21), further emphasizing non-overlapping roles of *in vivo* generated pTreg cells from those of tTreg cells.

Over the last decade, much attention has been paid to the tissue-resident T cell subset, a sessile population of antigen-experienced memory T cells within the non-lymphoid tissues. They play a critical role in protecting the host from pathogens reentering the tissue sites. They are also found to display transcriptionally distinct programs from those of circulating memory counterparts, suggesting a distinct mechanism for their homeostasis and functions (22, 23). It was subsequently reported that certain Treg cells also express tissue-resident properties especially first in the visceral adipose tissue (VAT), and then in numerous non-lymphoid tissues including the skeletal muscles, intestine, skin, and central nervous system. As we begin to understand more about distinct features of Treg cells residing in different tissue sites, this review will discuss various tissue-resident (TR)-Treg cell subsets and the cellular/molecular mechanisms shaping their homeostasis as well as their beneficial or pernicious functions.

## Treg Cell-Mediated Immune Suppression

The mechanisms through which Treg cells regulate immunity have extensively been examined over the years. Treg cell-mediated immune regulation is generally achieved *via* two modalities: contact-dependent and -independent mechanisms. Treg cells secrete copious amounts of anti-inflammatory/regulatory mediators to dampen inflammatory responses. IL-10 produced by Treg cells, although dispensable for tTreg cell development and not required to prevent systemic autoimmune inflammation, play an instrumental role in limiting inflammation especially at the mucosal interface (24). TGFβ1, a pleiotropic cytokine with potent immune suppressive property, is another cytokine produced by Treg cells (25). TGFβ1 produced by Treg cells was shown to be required to inhibit allergic and autoimmune inflammatory responses (26, 27), although the role of Treg cell-derived TGFβ1 has also been challenged (28). IL-35, composed of the IL-12p35 and Ebi3 subunits, is another Treg cell-derived cytokine known to antagonize T cell proliferation (29). Besides producing immune regulatory cytokines, Treg cells are also capable of suppressing effector T cell responses by consuming IL-2 (30). As for contact-dependent mechanisms, Treg cells express numerous inhibitory co-receptors capable of antagonizing immune activation. Lymphocyte activation gene 3 (Lag3) is a CD4-related receptor expressed by activated T cells, and Treg cells are known to express high level of Lag3 (31). Lag3-deficient Treg cells express deficient suppressive activity (31), although the precise underlying mechanism remains unclear. Lag3 may suppress antigen presenting cell maturation by interacting with its ligand, MHCII, thereby diminishing T cell activation (32). Cytotoxic T lymphocyte-associated protein 4 (CTLA4) is a B7 like molecule constitutively expressed in Treg cells and has been implicated in Treg cell suppression (33), although CTLA4-deficient Treg cells were still able to suppress immune responses (34). Treg cells can suppress effector T cell responses though degrading extracellular ATP. Treg cells highly express CD73 and CD39, ATP hydrolyzing ectoenzymes, on their surface. Adenosines and AMPs generated from degrading extracellular ATP have the capacity to inhibit antigen presenting cell maturation and T cell activation (35). In sum, the mode of suppressive mechanisms in Treg cells is likely determined by inflammatory and tissue conditions.

## Subsets Of Tissue Resident Treg Cells: Characteristics And Functions In Healthy/Disease Status

### Treg Cells in the Visceral Adipose Tissues (VAT)

While there were many reports describing Treg cells detected in tissues from the early era in the Treg cell field, the genesis of TR-Treg cell concept is a seminal study by Feuerer et al. that examined a unique Treg cell population within the VAT (36). First hint came from an unexpected observation that more than 50% of CD4 T cells found in the epididymal fat pads express Foxp3. These Treg cells are equally suppressive, and most importantly display transcriptional profiles that are distinct from Treg cells isolated from the counterparts of lymphoid tissues (36). VAT Treg cells are thought to be originated from the thymus. VAT Treg cells express high levels of Helios and neuropilin-1 (Nrp-1), markers considered for tTreg cells (37). TCR sequencing analysis uncovers little overlap between VAT Treg and conventional CD4 T cells from the lymphoid tissues (36, 37). Likewise, adoptively transferred conventional CD4 T cells do not give rise to VAT Treg cells (38). Notably, Treg cell accumulation in the VAT seems to occur early in life (prior to 3-4 weeks of age), since thymectomy at this age does not affect the numbers of VAT Treg cells (37). The contribution of circulating Treg cells in VAT Treg establishment seems minimal after 3 weeks as replenishing VAT Treg cells by adoptive transfer of Treg cells or using parabiosis model fails to do so (37).

Genes preferentially expressed in VAT Treg cells include transcription factors such as *Pparg*, *Rora*, and *Gata3*, and chemokines/cytokines and their receptors such as *Cxcl2*, *Cxcr6*, *Ccr1*, *Ccr2*, *Il10*, *Il5*, *Il1rl1*, and *Il9r* (39, 40). There are co-stimulatory receptors, *Pdcd1*, and *Ctla80*, and other proteins related to lipid metabolism including *Dgat1*, *Dgat2*, and *Cd36* highly expressed in VAT Treg cells (39, 40). Peroxisome proliferator-activated receptor (PPAR) is the master transcriptional regulator that controls adipocyte differentiation (41). It was found that Treg cell expression of the *Pparg* is essential for VAT Treg cell accumulation, phenotype, and functions (39). Treg cell-specific PPARγ-deficient mice show decreased VAT Treg cell number and loss of their function without affecting Treg cells from other tissues (39). Likewise, mice treated with a PPARγ agonist demonstrate a pronounced increase in the number of VAT Treg cells (39). In addition to the *Pparg* gene, the *Il1rl1* gene encoding ST2 protein, the receptor for IL-33, is also highly expressed in VAT Treg cells (39), suggesting that IL-33 may play a role in VAT Treg cells. Indeed, IL-33 promotes VAT Treg cell accumulation (37, 42). Single-cell RNA sequencing analysis showed trajectory of VAT Treg cells, as the splenic Treg cells expressing low levels of PPARγ contain precursors of TR-Treg cells, which accumulate in non-lymphoid tissue including VAT, further supporting a critical role of PPARγ in tissue adaptation of Treg cells (43).

One notable feature of VAT Treg cells is that they express a clonally expanded TCR repertoire (36, 37), strongly suggesting that there may be antigen(s) expressed within the tissue. By generating TCR Tg mice utilizing VAT Treg clone, Li et al. demonstrated that the Tg mice (named vTreg53) have substantial population of VAT Treg cells expressing the TCR. Consistent with the earlier transcriptional profiles from polyclonal VAT Treg cells, VAT Treg cells from the TCR Tg mice express representative gene signatures that are previously identified (38).

Obesity-associated metabolic diseases are in part driven by chronic inflammation of the VAT, and VAT Treg cells are central regulators of the inflammatory responses (36). In obese patients, the number of Treg cells in the circulation as well as VAT is substantially reduced (44). Eller and colleagues showed that significant decrease in the number of VAT Treg cells is closely associated with an increase in inflammatory mediators and a decrease in insulin sensitivity in VAT (45). In contrast, supplementation with Treg cells reduced VAT inflammation and improved metabolic parameters in obese mice (45). How VAT Treg cells are reduced during obesity has recently been explored. IFNα produced by plasmacytoid dendritic cells could deplete PPARγ+ VAT Treg cells (46). Alternatively, the soluble isoform of IL-33 receptor ST2 (sST2) acts as an obesity induced adipokine capable of antagonizing IL-33 signaling, thereby disrupting VAT Treg cell homeostasis (47).

Cold exposure is another factor that regulates VAT Treg cell accumulation. Short-term cold exposure enhances *in vivo* Treg cell induction in mice and humans by upregulating C17orf59 that inhibits mTORC1 signaling, thereby enhancing Treg cell generation (48). It has been reported that Treg cells are more enriched in brown adipose tissue (BAT) and subcutaneous adipose tissue (SAT) compared to VAT, implying the impact of cold exposure on generation and accumulation of Treg cells in fat tissues (49). Systemic ablation of Treg cells results in compromised energy expenditure adaptation upon cold exposure, accompanied with increased macrophage infiltration into brown adipose tissue (BAT) (50), indicating a novel role of Treg cells in thermogenesis. Treg cells upregulate thermogenic gene expressions upon cold exposure or β3-adrenergic receptor agonist CL316243 stimulation (50, 51). Interestingly, the role of SAT Treg cells in promoting adipocyte beiging and thermogenesis appears to be more prominent in female mice than male mice (51). This finding suggests a possible sex difference in the role of fat Treg cells. Along the line, the role of sex hormones in regulating VAT Treg cell accumulation has recently been uncovered, as androgen promotes production of IL-33 from stromal cells, which results in local expansion of VAT Treg cells in males (52). The precise contribution of sex hormones during fat Treg cell homeostasis needs to be investigated.

However, there is also emerging evidence that VAT Treg cells may negatively influence insulin resistance. IL-10 secreted from Treg cells, including VAT Treg cells, can drive insulin resistance in obesity by suppressing adipocyte energy expenditure and thermogenesis (53). In support, Treg cell specific IL-10 deficiency leads to increased insulin sensitivity and reduced obesity in male mice fed with high fat diet. Blimp1 is a transcription factor involved in IL-10 expression in Treg cells (54). Blimp1 deficiency in Treg cells reduces IL-10+ Treg cells especially within the adipose tissue, and Blimp1 deficient mice are protected from insulin resistance and obesity (53). Zheng and colleagues reported that VAT Treg cells may play a distinct role during insulin resistance derived from obesity and from aging (55). Indeed, mice deficient in VAT Treg cells, while susceptible to obesity associated insulin resistance, are better protected from age-associated insulin resistance (55), suggesting a complexity of VAT Treg cell-mediated regulation of insulin metabolism. A recent study from Li et al. uncovered using single-cell ATAC-sequencing that insulin signaling triggers the transition of VAT Treg cell subsets (CD73^hi^ST2^lo^ subset into a CD73^lo^ST2^hi^ subset) through a HIF-1α-Med23-PPARγ axis (56). In obese and aged mice, CD73^hi^ST2^lo^ VAT Treg cells are markedly decreased (56). In support, deleting Hif1α or Med23 in Treg cells is sufficient to block the transition, resulting in an enrichment of CD73^hi^ST2^lo^ VAT Treg cells, which activate beige fat biogenesis and protect from metabolic disorders (56).

Lastly, the discrepancy on adipose tissue TR-Treg cells among species is a subject of high importance. A study by Laparra et al. directly compared the proportion of Treg cells present in VAT or SAT among mouse models, cynomolgus macaques, and humans (57). Surprisingly, the proportion of fat TR-Treg cells is found low, except C57BL/6 strain which show male-specific and aging-related increase of Treg cells. As C57BL/6 mouse strains are widely used to study VAT Treg cells, this species/strain-related discrepancy needs to be taken into account when translating the findings to humans.

### Treg Cells in the Skin

Skin is the largest organ of the body and the importance of cutaneous Treg cells in maintaining tissue homeostasis and immune tolerance especially against skin microbiome has been investigated. In the skin of adult mice, the proportion of Foxp3^+^ Treg cells is considerably higher than that of circulation or of lymphoid tissues (~40% vs. ~10%) (58). Gratz et al. reported the existence of Treg cells expressing activated effector memory phenotypes that preferentially locate in hair follicles in the skin of mice (59). Memory phenotype Treg cells found in normal human skin are similarly localized in hair follicles (60). Ontogeny of skin resident Treg cells begins early at postnatal period, during which immune tolerance against commensal microbes is thought to be established (58). Preventing Treg cell accumulation in the skin by treating sphingosine-1-phosphate receptor agonist (FTY720) substantially disrupts tolerance against commensal bacteria, upon challenge later (58). A subset of CCR6^high^ Treg cells found within the neonatal thymus are thought to be those seeding the skin during postnatal period (61). Inhibiting the migration during this developmental window results in accumulation of these Treg cells in the thymus, suggesting that they are of thymic origin.

Skin Treg cells are highly heterogeneous. Comparing TCR sequences between conventional memory T helper cells and Treg cells isolated from human skin revealed little homology, indicating that they may recognize different antigens (60). An elegant study from Tomura and colleagues utilized a mouse model expressing a photoconvertible protein to track migratory cells during contact hypersensitivity. From single cell gene and protein expression analyses they identified two populations of skin Treg cells, one subset primarily expressing Nrp1 and another subset that express CD39 together with CD25, granzyme B, or CTLA4 (62). Skin Treg cells are also known to express GATA3 and RORα (63), and they may directly control Treg cell functions because mice deficient in these transcription factors in Treg cells spontaneously develop Th2 type skin inflammation (64).

Skin-specific environmental cues help to promote skin TR-Treg cell development and proper functions. Skin commensal microbes play an important role for the early recruitment of Treg cells to the skin, since germ-free neonates display substantial reduction in skin Treg cells (61). Microbiota are known to stimulate CCL20 production by hair follicles, supporting the migration of Treg cells (61). Phenotypic features of skin resident Treg cells are analogous to that of memory T cells in that they express low levels of CD25 yet higher KLRG1, CTLA4, and CD127 (59). Moreover, various chemokine receptors involved in skin migration are expressed, including CCR2, CCR6, CCR8, CCR10, CXCR4, and CXCR6. IL-15 and dermal fibroblasts, conditions found in chronically inflamed skin, trigger cocultured cutaneous Treg cell proliferation in an antigen-independent manner, indicating that skin Treg cells may directly respond to inflammatory cues to limit inflammatory processes (65). Likewise, IL-33, an alarmin cytokine highly expressed in the skin from patients with atopic dermatitis or psoriasis, is known to drive accumulation and functional maturation of skin Treg cells (66). Interestingly, unlike lymphoid Treg cells, skin Treg cells are found to highly express both IL-2 and IL-7 receptors (59). However, IL-2 is dispensable for their maintenance in the skin. Instead, IL-7 seems crucial for their retention in the skin at steady state (59). Additionally, skin Treg cells express the mitochondrial enzyme, arginase 2 (Arg2) (67). Arg2 augments suppressive functions of Treg cells in part by regulating their metabolic program *via* mTOR signaling (67). Arg2 deletion in primary human Treg cells results in the loss of Treg transcriptional signature (67), suggesting broad functions of Arg2 in Treg cells.

There is a plethora of evidence emphasizing the importance of skin Treg cells for maintaining tissue homeostasis. Skin Treg cells express Notch ligand family member, Jagged 1, and facilitates hair follicle stem cell function and hair follicle regeneration by supporting stem cell proliferation and differentiation through the CXCL5-IL-17 axis (68). It was recently reported that obesity targets hair follicle stem cells to accelerate hair thinning and loss (69). High-fat diet (HFD) induced IL-1-derived NFkB activation within the stem cells, which inhibits Sonic hedgehog (SHH) signal transduction in the stem cells, thereby inducing depletion of hair follicle and hair loss. Because HFD is known to impair Treg cell function especially in the fat tissues, it is interesting to speculate that HFD inhibits hair follicle stem cells through altering skin Treg cell functions. Skin Treg cells also inhibit myofibroblast activation, which suppress excessive scar formation during wound healing (64). Treg cells can promote the repair of skin trauma by the expression of epidermal growth factor receptor (EGFR) and are involved in stem cell differentiation in the skin (70). In support, the lack of EGFR in Treg cells can lead to delayed wound closure and increase accumulation of pro-inflammatory macrophages (70). Expansion of skin Treg cells with a healing function upon UVB exposure has been reported (71). The expanded Treg cells promote wound healing and suppresses inflammation, *via* proenkephalin (PENK), an endogenous opioid precursor, and amphiregulin (Areg), the EGFR ligand (71).

Skin Treg cells are fundamentally important in regulating inflammatory responses in the skin. In vitiligo, a T cell mediated autoimmune condition resulting in the loss of melanocytes, cutaneous Treg cells are greatly reduced, suggesting that autoreactive T cell activation is not adequately controlled (72). Diffuse systemic scleroderma/sclerosis (SSc) is an autoimmune condition characterized by excessive fibrosis in barrier tissues like skin (73). Skin Treg cells are similarly reduced in the SSc skin, which seems associated with diminished TGFβ and IL-10 expression (74). However, one study that compared Treg cells from blood and the skin of limited and diffuse SSc patients found that Treg cells in the skin lesions produced pro-fibrotic Th2 cytokines like IL-13 and IL-4 (75). Along the same line, an increase in the frequency of circulating CD25+FoxP3+CD127− Treg cells in SSc patients was observed especially in early phase of the disease. There is also recent evidence indicating that Treg cells could contribute to SSc pathogenesis by conversion into pathogenic effector T cells (76). Fenoglio et al. found a significant correlation between increased circulating Th17 cells and alteration of Treg cell compartment (77). Therefore, these dysfunctional Treg cells may exacerbate the disease. Elevated methylation levels of the FoxP3 promoter, inversely correlated with FoxP3 mRNA expression, are accompanied by reduced proportion of CD4+CD25+FoxP3+ Tregs (78). Indeed, increased methylation levels of the FoxP3 promoter in Ssc patients was found associated with reduced FoxP3 mRNA expression and proportion of CD4+CD25+FoxP3+ Treg cells (79). Treg cells are also able to limit psoriatic inflammation in the skin. Experimental psoriasis induced by topical imiquimod application results in significant accumulation of Treg cells in the skin lesion, as seen in human psoriasis (80). The accumulation is thought to mediate resolution of the inflammation, because Treg cell depletion dramatically exacerbates skin inflammation (80). However, Treg cells may alternatively promote the disease in some cases. The disease-promoting features of Treg cells are attributed to Treg cells’ differentiation into IL-17-producing effector-like cells (81, 82).

### Treg Cells in the Lung

As the lung is constantly exposed to microbial and environmental antigens, balancing tolerance and immunity is an important task of lung resident immune cells. The lung is another organ replete with TR-Treg cells. Treg cells resident in the lung tissue express various immune regulatory properties during infection, allergy, and injury. In asthmatic inflammation, Foxp3 expression in TR-Treg cells or the frequency of TR-Treg cells is found diminished (83), although in some cases, Treg cell numbers are instead increased in patients with severe allergic inflammation, suggesting their defective suppressive functions (84). The precise mechanisms underlying the defects is not well understood. However, inflammatory cytokines may destabilize the stability of Treg cells or undermine Treg cell functions. Thymic stromal lymphopoietin (TSLP) highly present in allergic conditions is shown to inhibit IL-10 production by Treg cells, resulting in diminished suppressive functions (85). Similar defective functions and reduced numbers of TR-Treg cells are found in idiopathic pulmonary fibrosis (IPF), a chronic lung disease caused by the accumulation of extracellular matrix in the lung (86). Earlier studies identified that Treg cells in the bronchoalveolar lavage are reduced in patients with IPF (87). Reilkoff et al. reported that Semaphorin (Sema) 7a expression is increased in lymphocytes including Treg cells and that Sema 7a^+^ Treg cells represent functionally defective Treg cells potentially supporting fibrosis in a TGFβ-driven fibrosis (88), suggesting that targeting Sema 7a+ Treg cells could be a strategy of therapy. In addition, an elegant study from Belperio and colleagues presented the evidence supporting a pathogenic role of Treg cells during fibrosis in the lung. Using bleomycin-induced fibrosis model in mice, treatment of mice with IL-2 complex during intratracheal bleomycin challenge exacerbates lung fibrosis, which was in part due to heightened type 2 immunity triggered by altered Treg cell functions (89). On the other hand, ST2^+^ Treg cells producing IL-13 in response to IL-33 are shown to be critical in preventing mortality following acute lung injury by limiting inflammatory cytokine production and Ly6C monocyte accumulation (90). Therefore, expanding Treg cells may deviate immune responses to type 2 immunity, yet the impact of type 2 immune responses remains controversial. Unlike bleomycin-induced lung injury model, CD103+ TR-Treg cells in the lung could potentially limit lung fibrosis induced by TR-pathogenic CD4 in Aspergillus infection model (91). Ichikawa et al. demonstrated that in response to chronic exposure to *Aspergillus fumigatus* CD69+CD103+ TR-Treg cells are generated to limit pathogenic functions of CD69+CD103+ TR-effector memory CD4 T cells to suppress fibrotic inflammatory responses (91). Therefore, the precise roles of TR-Treg cells may be determined by the type of immune responses in the lung.

The contribution of lung TR-Treg cells has also been explored in the model of lung infection. During pulmonary infection with Cryptococcus, antigen specific Treg cells are induced and accumulate in the lung. These Treg cells uniquely express the IRF4 to suppress Th2 type immune responses induced to control fungal replication and pathology. IRF4 expression in Treg cells plays a critical role for their retention in the lung through CCR5 expression (92). In viral infection such as influenza virus, antigen specific Treg cells expressing memory phenotype accumulate in the lung tissue and control anti-viral responses (93). Helios, an Ikaros family member transcription factor and preferentially expressed in Treg cells, was recently reported to be highly expressed in lung Treg cells during influenza virus infection. Importantly, Helios+ Treg cells have the ability to persist in the lung tissues and to more potently suppress virus specific CD8 T cell responses (94). Treg cells are also shown to mediate rapid antigen-specific primary and memory T cells responses (95) and to promote formation of follicular helper T cell and germinal center B cell responses in the lymph nodes by controlling IL-2 availability (96), thereby enhancing anti-viral immune responses. In addition to modulating anti-viral immunity, lung Treg cells promote tissue repair. Arpaia et al. previously reported that inflammatory cytokine IL-18 or alarmin IL-33 is able to induce Areg production from lung Treg cells during lung injury from viral infection, and the production of Areg seems crucial for Treg cell-mediated tissue repair (97). Given that TCR- and IL-2-signaling pathways primarily control Treg cell suppressive function, this study suggests that a distinct pathway triggered by ‘innate’ tissue-derived signals generated during inflammation and tissue injury is critically involved in tissue repair function, independent of the Treg cell suppressive function. Transcriptomic analysis of human TR-Treg cells uncovers that human lung TR-Treg cells differentially express genes associated with the Wnt pathway (specifically Wnt ligands, wnt1, wnt2, wnt7a and Wnt receptor, Fizzled 2), supporting the possibility that lung TR-Treg cell may mediate epithelial cell repair and regeneration (98).

Amid the SARS-CoV2 pandemic, the roles of lung TR-Treg cells during COVID-19 pathogenesis remain to be determined. Circulating Treg cell number is greatly reduced in COVID-19 patients, and importantly, the reduction rate is correlated with disease severity (99–102). A recent study also reported a decrease in airway Treg cells in COVID-19 patients compared to healthy controls (103). The loss of Treg cells in circulation seems related to increased and persistent tissue damage, which often leads to worsen disease outcomes. On the other hand, another study by Galvan-Pena et al., showed increase in proportions and Foxp3 levels in circulating Treg cells, correlating with disease severity (104). These discrepant findings are likely attributed to lung TR-Treg cell heterogeneity, suggesting a need for more precise characterization of lung TR-Treg cells beyond the change in their number or ratio. Evaluating antigen specificity of Treg cells to virus will be necessary based on the protective role of antigen-specific CCR7+ tissue resident memory (Trm) CD8 T cells in the non-hospitalized and convalescent patients, emphasizing the importance of persistent antigen-specific Trm cells (105). Given that lung TR-Treg cells can limit pulmonary immunopathology during respiratory virus infection, understanding the behavior of lung TR-Treg cells from COVID-19 patients may provide better insights into the understanding the pathology of this virus. However, a study from Benoist and colleagues reports that Treg cells from severe COVID-19 patients appear to express distinct phenotypes, unexpectedly characterized by increased Treg cell proportion and Foxp3 expression (104). It was found that these Treg cells overexpress not only suppressive effectors but also pro-inflammatory cytokine such as IL-32. Most strikingly, these Treg cells express similar traits as tumor-infiltrating Treg cells. These results suggest a detrimental role of Treg cells in COVID-19, by interfering with anti-viral immunity (104).

### Treg Cells in the Skeletal Muscle

Treg cells present in the skeletal muscle were first reported in 2013 (106). A small proportion of Treg cells (~10% of CD4+ T cells) exist in the muscle of healthy mice (106). In the injured condition, Treg cells rapidly proliferate within 3-4 days and then decrease. However, even after a month after injury, detectable Treg cells remain (106, 107). Accumulation of Treg cells in injured muscle is primarily mediated by IL-33 secreted by injured skeletal muscle (107). IL-33 acts on the cells expressing IL33 receptor, ST2, one of the most strongly up-regulated genes in Treg cells isolated from injured muscle compared to Tregs present in lymphoid tissue (106).

Where these Treg cells are originated from remains unclear. Treatment of FTY720 before inducing muscle injury reduced the absolute number of accumulated Treg cells, but not their proportion among CD4+ T cells in the muscle (107), suggesting accumulation of muscle Treg cells in response to injury may be dependent on the recruitment from the circulating T cell pool. However, this does not rule out the possibility that local expansion of Treg cells that are already found in the muscle. The TCR sequences of muscle Treg cells were distinct from muscle conventional T cells and a substantial proportion (20-40%) of muscle Treg cells is clonally expanded (106). Using a transgenic mice carrying rearranged transgenes encoding the TCRα and TCRβ chains from a muscle TR-Treg clone, Cho et al. showed a muscle specific accumulation and proliferation of Treg cells, indicating TCR-dependent development of muscle Treg cells (108). Therefore, muscle Treg cells may arise from the mixed origins, and this needs to be further examined.

Skeletal muscle regeneration requires the activation and differentiation of myogenic stem cells, called muscle satellite cells (109). Upon muscle injury, proinflammatory (M1 type) macrophages immediately infiltrate the tissue, followed by anti-inflammatory (M2 type) macrophages (110). M1 macrophages stimulate satellite cell proliferation and exacerbate inflammation, while M2 macrophages induce differentiation of satellite cells and promote tissue regeneration (110). Muscle Treg cells play a crucial role in M1-to-M2 switch in macrophages as this transition is absent when Treg cells are depleted (106). Treg cells also limit IFNγ production, reducing inflammation and fibrosis (111). Furthermore, Treg cells may directly regulate activity of muscle satellite cells. *In vitro* coculture with Treg cells induces enhanced proliferation and reduced myogenic differentiation of muscle satellite cells (106). Regulatory role of Treg cells on tissue regeneration seems to be dependent on a growth factor, Areg. Areg is overexpressed by muscle Treg cells and administration of Areg to Treg-depleted mice at the time of muscle injury induces expression of genes involved in muscle repair (106). Addition of Areg in the muscle satellite cell culture also enhances differentiation of myogenic differentiation (106), suggesting a critical role of Areg, particularly derived from Treg cells, on muscle repair.

Little is known about Treg cells in muscle disease. Treg cells were highly accumulated in skeletal muscles of *mdx* dystrophin-deficient mice, a model of human Duchenne muscular dystrophy (DMD), in which muscle injury and inflammation is mitigated by expansion of the Treg population but exacerbated by Treg-cell depletion (112). The P2X7 purinergic receptor (P2RX7) appears to be especially important in this respect. Genetic ablation of the P2X7 gene in *mdx* mice or P2RX7 antagonist administration leads to improved muscle structure and function, with reduction of inflammation and fibrosis, and increased muscle strength and endurance (113, 114). Muscle Treg cells also support muscle repair after myocardial infarction directly *via* promoting cardiomyocyte proliferation (115) and indirectly *via* modulating M1/M2 differentiation (116). More work is needed to understand TR-Treg cell biology in the muscle.

### Treg Cells in the Intestine

The gut tissues are separated from microbial and food antigens by single layer of intestinal epithelial cells (117). Therefore, the epithelial layers as well as underlying tissues, lamina propria, are heavily populated with various immune cells particularly with regulatory properties. In support, the intestines are most frequently affected by the loss-of-function mutations of the *FOXP3* gene in IPEX patients, highlighting the key role of Treg cells in the intestinal tolerance (118). Intestinal TR-Treg cells are heterogeneous and can be divided into the three subsets based on transcription factor expression: GATA3+Helios+, RORγt+, and RORγt-Helios- Treg cells (119). Wang et al. reported that Treg cell-specific deletion of GATA3 results in a spontaneous inflammatory disorder (120). These Treg cells display defective suppressive function with diminished Foxp3 expression, instead acquire Th17 phenotypes (120). In contrast, Wohlfert et al. reported that Treg cell-specific Gata3-/- mice do not exhibit any inflammatory conditions at steady state. However, the frequency of Treg cells in the intestinal tract is found markedly diminished in these mice (121). Consistent with this observation, Gata3-/- Treg cells fail to protect animal from colitis (121). Particularly, GATA3 expression in Treg cells plays an important role in limiting expression of effector T cell markers such as RORγt and IL-17A especially under inflammatory settings (121). GATA3 also induces expression of ST2 and loss of GATA3 markedly reduces ST2 expression in Treg cells (122). RORγt, a transcription factor involved in Th17 cell differentiation, is also found highly expressed in colonic Treg cells compared to splenic Treg cells at steady state and its expression is microbiome-dependent (123, 124). RORγt supports Foxp3 expression in colonic Treg cells in part by suppressing the effector programs (125). RORγt-deficient Treg cells transferred into Rag-/- mice induced for colitis fail to protect mice from the disease, as these Treg cells express Th1-like effector phenotypes and subsequently lose suppressive activity (125).

Intestine TR-Treg cells likely have mixed origin. Approximately 10% of CD4+ T cells express Foxp3 in germ-free mice, while ~35% of colonic CD4+ T cells are Treg cells in specific pathogen-free (SPF) mice (126), suggesting that the majority is likely of locally generated pTreg cells. Indeed, analysis on colonic Treg cells in SPF mice showed low expression of Helios and Nrp-1, markers of tTreg cells (107, 126, 127). Adoptive transfer of naïve conventional T cells from lymph node (LN) to Treg-depleted mice successfully restored RORγt+ or Helios+ colonic Treg cells upon interaction with microbiota (128), indicating microbiome-derived signals are required for inducing certain subset of Treg cells in the gut. However, some Treg cells are likely of thymic origin. In support, TCR repertoire of intestine TR-Treg cells is similar to LN Treg cells but distinct from intestinal conventional T cells (129).

Inflammatory bowel disease (IBD) is a group of chronic inflammatory diseases of the intestine, including ulcerative colitis and Crohn’s disease. IBD patients have increased Treg cells in the mucosa of gut; however, the suppressive function of Treg cells from IBD patients seems impaired (130). Increased expressions of Tbet and RORγt are associated with elevated levels of proinflammatory cytokines including IL-17, IL-6, and IL-1β, resulting in a decreased suppressive Treg cell function and severe inflammation in IBD (131–133). Areg is also highly expressed by intestinal Treg cells; however, Treg-specific ablation of Areg does not alter the phenotypes nor functions of Treg cells, suggesting dispensable role of Treg cell-derived Areg in the intestine (97). Dysregulated Treg cell functions appear to contribute to the pathogenesis, although the precise role of dysregulated Treg cells in IBD etiology remains to be determined.

Treg cells play an opposite role in the context of colorectal cancer (CRC), where an increased accumulation of Treg cells is associated with worse prognosis and Treg cells suppress anti-tumor effector T cell immunity (134). However, it is also possible that Treg cells can be protective in CRC as Treg cells are the major source of IL-10, which reduce polyposis (135). Oral administration of IL-10 to a mouse model of spontaneous gastrointestinal polyposis ameliorates disease and enhances survival, *via* regulating the ratio between RORγt+IL-17+ and RORgt-IL-17- Treg cell subsets (136). This opposite role of Treg cells might be due to their extremely high heterogeneity in intestines. Single-cell RNA sequencing analysis on intratumoral Tregs from CRC patients revealed Treg cells with low activity of the MondoA-thioredoxin-interacting protein (TXNIP) axis and increased glucose uptake (137). Treg cells with low MondoA-TXNIP axis are Th17-like and hyperglycolytic, which promote tumor growth. This seems to be a result of adaptation to glucose-depleting tumor microenvironment. It was recently reported that TCF-1 plays an essential role in regulating Treg cell development and functions (138). Interestingly, TCF-1-deficient Treg cells, while fully competent to suppress T cell proliferation and cytotoxicity, were unable to control CD4 T cell polarization and inflammation (138). In mice with polyposis, the lack of TCF-1 expression in Treg cells promotes tumor growth. Likewise, in CRC patients TCF-1^low^ Treg cells and elevated Th17 immunity were found (138). In a mouse model of environmental enteric dysfunction (EED), a gastrointestinal inflammatory disease caused by malnutrition and chronic infection, Hand and colleagues recently reported that mice with EED harbor increased RORγt+ Treg cells in the small intestine, contributing to poor efficacy of oral vaccines (139). Deletion of RORγt expression in Treg cells was sufficient to restore small intestinal vaccine specific CD4 T cell responses and vaccine induced protection, suggesting a detrimental role of Treg cells. However, the lack of these Treg cells was associated with enhanced susceptibility to EED (139). These findings highlight the complex and diverse mechanisms mediated by intestine TR-Treg cells in different disease settings.

### Treg Cells in Tumors

?A3B2 twb .24w?>Treg cells are abundant in tumor tissues. It is generally known that intratumoral Treg cells hinder effective anti-tumor immunity, representing a critical barrier to successful anti-tumor immunity and immunotherapy. De Simone et al. compared Treg cells infiltrating colorectal or non-small cell lung carcinomas and found that intratumoral Treg cells are highly suppressive compared to Treg cells isolated from normal tissues or the peripheral blood of the same patients (140). Phenotypically, these Treg cells express immune checkpoint surface receptors (Lag3, CTLA4, PD1, TIM3). Expression of tumor Treg cell signature genes, such as *Layn*, *Mageh1*, and *Ccr8*, is found negatively correlated with poor patient survival (140). Similar features of intratumoral Treg cells are also found in human breast cancer, namely, high suppressive ability and their gene expression pattern, notably CCR8 (141). In prostate cancer patients, CCR4+ Treg cells are found abundant in the tumor tissue and associate with poor prognosis (142). Tumor microenvironment secretes chemokines selectively recruiting Treg cells to tumor sites. Hypoxia induces CCL28 in ovarian cancer and liver cancer, recruiting CCR10+ Treg cells (143). Pancreatic ductal adenocarcinoma secretes CCL5 to recruit CCR5+ Treg cells and blocking CCL5 expression could improve anti-tumor immunity (144). Anti-CCR4 antibody treatment enhances antitumor immune responses mainly by altering tumor infiltrating Treg cells in an ovarian cancer model (145). Similarly, anti-CCR4 antibody selectively depletes intratumoral Treg cells, promoting anti-tumor T cell responses (146).

TCR repertoire analyses uncover little overlap between effector T cells and intratumoral Treg cells in human breast cancer and melanoma patients (141, 147). Instead, tumor Treg cells display substantial overlap with circulating Treg cells, suggesting that they are likely of tTreg cell origin (147). Alternatively, tumor microenvironment can promote the differentiation of pTreg cells from naïve CD4+ T cells due to enriched signals including TGF-β, IL-10, and indoleamine 2,3-dioxygenase (IDO), all of which are known Treg cell inducers. For example, acute myeloid leukemia cells express IDO, supporting the intratumoral Treg cell conversion (148). The precise origin of intratumoral Treg cells may thus differ depending on the tumor cell types.

Treg cells accelerate immune evasion by tumor cells, leading to tumor development and progression in various types of cancer. Treg cells known to restrict the anti-tumor immune response through multiple mechanisms such as CTLA4 mediated suppression of antigen presenting cell function, consumption of IL-2, production of immunosuppressive cytokines, and expression of immune checkpoint inhibitory receptors. Lag3 is highly expressed in intratumoral Treg cells. Lag3 expressed on Treg cells was initially reported to play an important role in mediating Treg cell functions (31). Paradoxically, Lag3 expression in Treg cells was shown to limit Treg cell function and survival in a mouse model of autoimmune diabetes (149). Whether Lag3 expression in intratumoral Treg cells plays positive or negative role in Treg cell functions need to be examined. If Lag3 is an inhibitory receptor interfering with Treg cell function as observed in diabetes, targeting Lag3 in intratumoral Treg cells will enforce the function, further limiting anti-tumor immunity.

### Treg Cells in Transplantation

Treg cells derived from recipients are generally considered critical in preventing allograft rejection, as a selective depletion of recipient Treg cells but not donor Treg cells abrogates graft acceptance (150, 151). Using a murine cardiac allograft model, Hancock and colleagues found that ~100-fold higher intragraft Foxp3 levels in recipients tolerized with anti-CD154 antibody and donor-specific transfusion, compared to those rejecting allograft (152). Treg cells are further found indispensable for tolerance induction, because anti-CD25 antibody injection or peritransplant CD25+ Treg cell depletion prevents long-term graft survival (152). Similar protective roles of Treg cells were also reported in liver and lung transplantation (153, 154). In case of kidney transplantation, allografts are spontaneously accepted, which is associated with increased Foxp3 expression (155). Interestingly, donor-derived TR-Treg cells were more potent than the recipient-derived Treg cells (156). The precise mechanisms supporting the formation of TR-Treg cells and the cellular and molecular signatures of TR-Treg cells within different transplant organs remain to be determined.

## Crosstalk Between Treg Cells And Tissues

**Figure 1:**
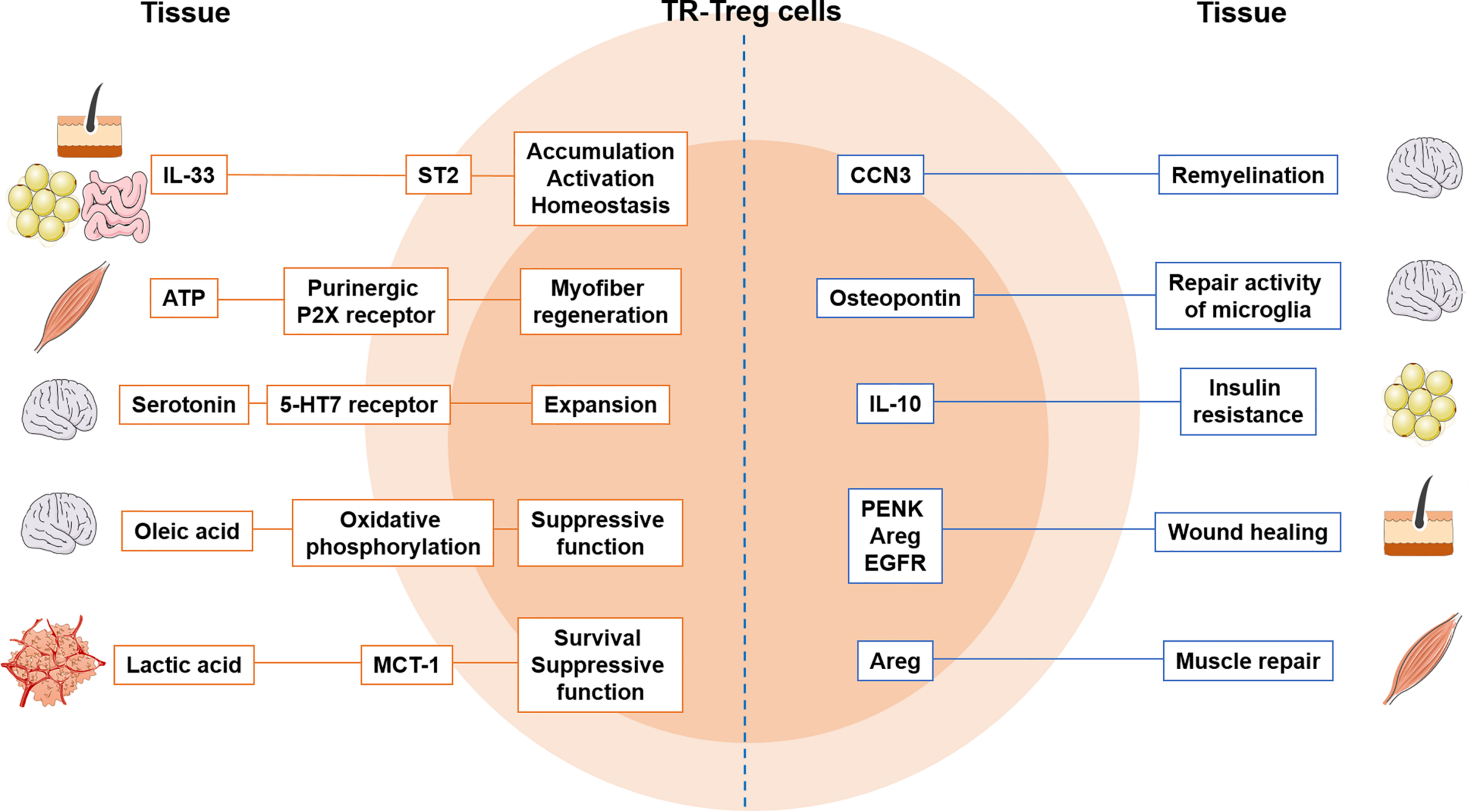
Tissue-specific adaptation of TR-Treg cells and crosstalk between tissue and Treg cells. The factors produced from tissues shape Treg cells’ ability to control in the tissue. Treg cells express markers to become more tissue specific and produce factors that regulate tissue function. MCT-1, lactate transporter; Areg, amphiregulin; 5-HT7, serotonin receptor; CCN3, cellular communication network factor 3; PENK, proenkephalin; EGFR, epidermal growth factor receptor.

Once activated Treg cells enter the inflamed or infected target tissues, they receive additional tissue-derived signals that allow them to further differentiate into TR-Treg cells. These signals are tissue-specific, and likely turn on the ‘tissue-specific’ signatures on Treg cells (**Figure 1**). For example, IL-33, a member of the IL-1 family and an alarmin, is mainly produced by epithelial and endothelial cells following damage or cell stress and function as a ‘pan-TR-Treg cell factor’ as they are expressed in multiple tissue sites (157). A study by Shiering et al. has found that IL-33 produced by inflamed intestines acts on colonic Treg cells that highly express the ST2 and supports Treg cell function and, most importantly, provides a signal for Treg cell accumulation and maintenance in the sites (122). Similar mechanism was also observed in intestinal cancer. Pastille et al. demonstrated that tumor infiltrating Treg cells preferentially express ST2 and that IL-33 stimulated intratumoral Treg cells display more activated phenotypes and better accumulate in the tumor (158). IL-33 also shapes Treg cell stability by curtailing IL-17 production by Treg cells. IL-33’s ability to modulate Treg cell homeostasis is also found in VAT. Vasanthakumar et al. reported that IL-33 expressed in the VAT is able to induce VAT TR-Treg cell proliferation and maintain Treg cell identity, because both proportions and Foxp3 expression of VAT TR-Treg cells are greatly diminished in Myd88-/- mice (159). Expression of the *Pparg*, a master transcription factor essential for VAT TR-Treg cells, is maintained through IL-33 stimulation, further supporting the IL-33’s support for VAT Treg cells (159). Last example of IL-33 function as a regulator of TR-Treg cells is from skeletal muscle. Kuswanto et al. demonstrated that IL-33 is rapidly produced after acute muscle injury and that IL-33 is primarily produced by CD31+ endothelial cells (107). IL-33 signaling is particularly important for muscle TR-Treg cell accumulation, as Treg cell-specific ST2-deficient mice exhibit delayed muscle repair (107). Most notably, tissue regenerative activities of Treg cells, for instance to induce tissue repair factors such as *Myog* (muscle transcription factor) and *Adam8* (constructing extracellular matrix) are significantly decreased in the absence of ST2 on Treg cells (107). In addition to soluble mediators like cytokine IL-33, there are tissue-specific mechanisms involved in TR-Treg cell trafficking, accumulation, and retention. FucT-VII (alpha-1,3-fucosyltransferase VII) is an enzyme required for PSGL-1 glycosylation to facilitate TR-Treg cell accumulation in tissues like skin and lamina propria (160, 161). ATP released by necrotic muscle cells during muscle injury inhibits TR-Treg cell generation and functions through purinergic P2X receptors, delaying myofiber regeneration (113, 114). In the CNS, serotonin (5-HT) produced by brain cells activates 5-HT7 receptor expressing Treg cells, supporting Treg cell expansion (162). Meanwhile, Treg cells infiltrating the brain during ischemic injury release osteopontin that enhances the repair activity of microglia (162). A protein known as cellular communication network factor 3 (CCN3) is another cellular factor secreted from CNS TR-Treg cells, promoting oligodendrocyte differentiation and remyelination (163).

The evidence that local metabolites critically modulate Treg cell functions continues to rise (164, 165). Treg cells heavily rely on oxidative phosphorylation for their homeostasis and functions (165, 166). Hafler and colleagues recently reported that tissue-derived lipid molecule plays a key role in maintaining Treg cell homeostasis (167). Oleic acid, the most prevalent free fatty acid, amplifies Treg cell-oxidative phosphorylation by increasing Foxp3 expression as well as Stat5 phosphorylation, enhancing Treg cell suppressive functions. Indeed, Treg cells isolated from multiple sclerosis (MS) patients express different transcriptomic signatures that resemble those exposed to proinflammatory arachidonic acid (167). In support, the concentration of oleic acid was significantly reduced, and exposing MS patient Treg cells to oleic acid restores defects in suppressive functions. Tumors provide hostile environments for recruited immune cells (metabolite-depleted, hypoxic, and acidic conditions). An elegant study by Delgoffe and colleagues recently reported that intratumoral Treg cells unexpectedly utilize glycolysis by-product, lactic acid as an alternative energy source (168). Treg cell expression of a lactate transporter MCT-1 is thus essential for Treg cell survival and function within tumors, as Treg cell specific MCT1 deletion results in slow tumor growth, and more importantly, in an increased response to immunotherapy. How metabolic rewiring of intratumoral Treg cells favors Treg cell proliferation, survival, and suppressive functions will require further investigation. Therefore, tissue-specific metabolites may play a fundamental role in modulating Treg cell homeostasis and functions by reprogramming gene expression profiles of TR-Treg cells to equip them with cellular machineries ideal for their adequate maintenance.

## Therapeutic Interventions

Therapeutic approaches targeting Treg cells have gained much attention and multiple clinical trials are underway to apply preclinical findings in clinical settings (**Table 1**). Strategies leveraging Treg cells vary depending on the disease type, as augmentation of Treg cell number and function is critical for autoimmune diseases whereas depleting or suppressing Treg cells is required for successful cancer treatment. Depletion of Treg cells has been traditional attempts predominantly by targeting CD25. The approach has expanded to other Treg cell markers such as CD122, CD44, 4-1BB, and CCR8 in order to improve the specificity of treatments (192). Recent studies suggest a new approach rather to weaken Treg cell function *via* stimulating costimulatory molecules such as OX40 and GITR (193, 194). Now that TR-Treg cells may acquire tissue-specific signatures in phenotypes and gene profiles, developing more specialized tools targeting tissue-specific TR-Treg cells, particularly in tumors of different tissue sites, will be needed. Combining Treg cell target approaches with other immunotherapies such as anti-PD1 and anti-PDL1 therapies has also been attempted in cancer patients. Of note, depleting Treg cells may trigger compensation program such as an increased infiltration of macrophage in tumor or a reduction in conventional T cells (195, 196), reducing the efficacy of immunotherapy. Therefore, modulating Treg cells has to be performed in consideration of multiple Treg cell-extrinsic factors.

**Table 1:** Therapeutic strategies targeting Treg cells.

	*Target*	*Drug*	*Clinical trials (NCT number)*	References
*Depletion/* *Suppression*	*CD25*	*Denileukin diftitox (Ontak)*	*FDA approved for cutaneous T cell lymphoma*	(169)
		Daclizumab	FDA approved for multiple sclerosis	(170)
		LMB-2	Adult T cell lymphoma (NCT00924170)	(171)
	CD122	Bempegaldesleukin	Advanced solid tumor (NCT03435640)	(172)
	CD44 and 4-1BB	Depleting antibody	Sarcoma (NCT03282344), melanoma (NCT03635983)	(173)
	OX40	Depleting antibody	Preclinical	(174)
		Agonist antibody	Head and neck squamous cell carcinoma (NCT02274155), advanced solid tumors (NCT02737475)	(175, 176)
	GITR	Agonist antibody	Advanced solid tumor (NCT02583165, NCT02598960)	(177, 178)
	CCR4	Mogamulizumab	FDA approved for cutaneous T cell lymphoma	(179)
		Diphtheria toxin-based anti-human CCR4 immunotoxin	Preclinical	(180)
	CCR8	Depleting antibody	Preclinical	(181, 182)
*Promotion*	CD25	low dose IL-2	T1D (NCT01862120), SLE (NCT02465580), Autoimmune diseases (NCT01988506)	(183–185)
	mTOR pathway	Rapamycin (Sirolimus)	FDA approved for kidney transplantation and lymphangioleiomyomatosis	(186, 187)
*Cell therapy*		Autologous polyclonal expanded Treg cells	T1D (NCT02691247), Crohn's disease (NCT03185000), pemphigus (NCT03239470)	(188, 189)
* *		Engineered CAR-Treg cells	kidney transplantation (NCT04817774)	(190, 191)

Likewise, CD25 can also be a target to promote Treg cell expansion as treatment with low dose IL-2 or IL-2:anti-IL-2 complexes that selectively expand Treg cells including TR-Treg cells (197). However, IL-2 can still be used by CD25+ effector T cells, or TR-Treg cells in different tissues may utilize other factors (e.g. IL-7 or TGFβ) to maintain in certain tissues. Therefore, more specialized and tailored approaches to different tissues are necessary to support Treg cell homeostasis.

## Conclusion and Outstanding Questions

It is obvious that TR-Treg cells are critical regulators of tissue functions including homeostasis, inflammation, and repair, or a significant barrier antagonizing effective anti-tumor immunity. Over the years, the field has significantly grown and identified TR-Treg cells within different tissue sites, primarily skin, muscle, and VAT. We now begin to understand the origin of TR-Treg cells and key tissue-derived factors that promote the generation and maintenance of TR-Treg cells. There are several important questions that warrant future investigations. First, we need to better understand the mechanisms involved in TR-Treg cell retention within the tissues. Tissue retention is an important feature of tissue resident lymphocytes and appears to be mediated by multiple mechanisms depending on the tissue types and the nature of immune responses. Treg cell retention at the site of Leishmania infection is in part mediated by CD103, the αE chain of the αEβ7 integrin that is highly expressed on Treg cells (198). Sphingosine 1-phosphate receptor 5 (S1PR5) has recently been identified as a key regulator of the peripheral retention of TR-memory CD8 T cells (199). TR-Treg cells may share similar mechanisms for efficient tissue retention. Second, there is increasing evidence that local tissue antigen stimulation is critical for TR-Treg cell maintenance. A TCR transgenic mice specific for VAT-specific antigens has opened new opportunity to understand VAT TR-Treg cell biology (38). More rigorous TCR repertoire analyses and identification of key tissue specific antigens supporting TR-Treg cell differentiation will obviously be the next step.

## Author Contributions

JL, DK, and BM wrote the manuscript. All authors contributed to the article and approved the submitted version.

## Funding

This work was supported by NIH grant AI125247 and NMSS grant RG1806-31374 (BM).

## Conflict of Interest

The authors declare that the research was conducted in the absence of any commercial or financial relationships that could be construed as a potential conflict of interest.

## Publisher’s Note

All claims expressed in this article are solely those of the authors and do not necessarily represent those of their affiliated organizations, or those of the publisher, the editors and the reviewers. Any product that may be evaluated in this article, or claim that may be made by its manufacturer, is not guaranteed or endorsed by the publisher.
